# N6-Methyladenosine (m6A) RNA Modification in Periodontitis: Molecular Mechanisms and Therapeutic Potential: A Narrative Review

**DOI:** 10.3390/genes17101228

**Published:** 2026-10-03

**Authors:** Filippos Fytros, Stefanos Zisis, Vasileios Zisis, Petros Papadopoulos, Christina Charisi, Konstantinos Poulopoulos, Panagiota Dimitropoulou, Nikolaos Shinas, Joe Cordahi, Athanasios Poulopoulos

**Affiliations:** 1 Department of Dentistry, School of Dentistry, European University Cyprus, Nicosia 2404, Cyprus; filipposfytros04@gmail.com (F.F.);; 2 Clinic for Operative Dentistry, Periodontology and Preventive Dentistry, University Hospital RWTH Aachen, 52074 Aachen, Germany; 3 Department of Oral Medicine and Pathology, School of Dentistry, Aristotle University of Thessaloniki, 54124 Thessaloniki, Greece; 4 Department of Dentoalveolar Surgery, Surgical Implantology and Radiology, School of Dentistry, Aristotle University of Thessaloniki, 54124 Thessaloniki, Greece; 5 Department of Oral and Maxillofacial Radiology, Henry M. Goldman School of Dental Medicine, Boston University, Boston, MA 02118, USA; nshinas95@gmail.com (N.S.);

**Keywords:** N6-methyladenosine, m6A, periodontitis, METTL3, METTL14, FTO, ALKBH5, epitranscriptomics, periodontal ligament stem cells, inflammation, osteogenesis, alveolar bone loss

## Abstract

Background: N6-methyladenosine (m6A) is the most abundant mRNA internal modification in eukaryotes and regulates inflammatory and regenerative processes. Many studies relate the dysregulation of m6A-related pathways to periodontal inflammation, imbalance in cell homeostasis, impaired tissue regeneration, and remodeling of the alveolar bone. However, the mechanisms of the relationship between the m6A pathway and periodontitis have not been elucidated clearly yet. Objective: The objective of the current narrative review is to integrate the information related to the role of m6A RNA modification in the pathogenesis of periodontitis by focusing on inflammatory signaling, osteoimmune regulation, periodontal cell activity, bone remodeling, and its possible applications as a diagnosis and treatment approach. Methods: A literature search was conducted in PubMed to identify original research studies investigating the role of m6A RNA methylation and its regulators in periodontitis and periodontal tissues/cells. Out of 49 publications reviewed, 34 original research articles fulfilled the predetermined inclusion criteria. Results: The current literature suggests that N6-methyladenosine (m6A) modification is involved in various interconnected biological processes contributing to the development of periodontitis, including inflammatory responses, inflammasome activation and pyroptosis, osteoimmune reactions by macrophages, osteogenic differentiation, osteoclast formation, cellular senescence, and periodontal cells homeostasis. METTL3 has been reported to be the most widely investigated regulator, but its roles are not consistently harmful or beneficial. Depending on the RNA target, cellular environment, and state of inflammation, some of the m6A-associated pathways have been linked to periodontal inflammation and alveolar bone resorption, while others were linked to osteogenesis and regeneration. Several studies have highlighted the possibility of therapeutic interference in pathways leading to the formation of m6A, while human transcriptomic and gingival/salivary studies suggested that m6A could be used as a biomarker. Conclusions: The m6A modification of RNA is an emerging epitranscriptomic effect relating periodontal inflammation, immune responses, cellular differentiation, and bone remodeling. Even though target molecules and signaling pathways have been experimentally identified as points of potential therapeutic interference, the available evidence remains predominantly preclinical. Future work is expected to validate the diagnostic and therapeutic involvement of the m6A modification in periodontitis.

## 1. Introduction

Periodontitis is a chronic, multifactorial inflammatory disease characterized by progressive destruction of the tooth-supporting tissues, including the periodontal ligament and alveolar bone, potentially resulting in attachment loss and tooth loss long term [1,2]. The onset of periodontitis is stimulated by the action of periodontal pathogenic microorganisms with the dysregulated host-inflammatory and immunological reactions playing a leading role in the development of pathology, where the cytokine production leads to connective tissue destruction and bone resorption [1,2]. One of the major pathogens implicated in periodontal diseases is *Porphyromonas gingivalis* and lipopolysaccharide (LPS), which is one of its virulence factors, is often used to create a similar inflammatory environment in experimental settings [3]. Periodontitis therefore represents a complex interaction between microbial, environmental, inflammatory, and host-related factors that ultimately determines the balance between periodontal tissue destruction and repair [2].

Increasing attention has been directed toward epigenetic mechanisms capable of linking environmental and inflammatory stimuli with alterations in gene expression during periodontal disease [1,2]. Epigenetic regulation influences gene activity without altering the underlying DNA sequence and includes mechanisms such as DNA methylation, histone modifications, non-coding RNAs, and RNA modifications [1]. Advances in the science of molecular biology have expanded the scope of study on epigenetics to cover the area of epitranscriptomics, which includes chemical modifications of RNA and its effects on the regulation of post-transcription genes [4]. Of all chemical RNA modifications, N6-methyladenosine modification is now seen as one of the most prevalent internal modifications of eukaryotic messenger RNA and regulates multiple cellular functions [5,6].

The m6A modification of mRNA is a dynamic and reversible process that involves the activities of three major classes of proteins: writers, erasers, and readers [5,7]. Writers such as methyltransferase-like 3 (METTL3), methyltransferase-like 14 (METTL14), and Wilms tumor 1-associated protein (WTAP) are involved in the deposition of m6A marks, whereas fat mass and obesity-associated protein (FTO) and AlkB homolog 5 (ALKBH5) are some of the proteins responsible for the removal of these marks through the mechanism of demethylation [5,6]. Reader proteins, including members of the YTH domain-containing and insulin-like growth factor 2 mRNA-binding protein (IGF2BP) families, recognize m6A-modified transcripts and mediate their downstream biological effects [6]. Through these actions, m6A modifications affect RNA stability, RNA splicing, nuclear export, RNA degradation, and RNA translation, thereby influencing multiple physiological and pathological cellular processes [6,7].

A number of studies provide evidence that disturbance in the m6A regulatory pathway may contribute to periodontal inflammation and the progression of periodontitis [8,9]. Transcriptomic and epitranscriptomic analyses have identified altered expression of m6A regulators and distinct methylation patterns in periodontitis, some of which have been associated with immune-cell infiltration and inflammatory responses [8,9,10]. In addition, the regulators of m6A may alter inflammatory signaling pathways relevant to the destruction of periodontal tissues like the NF-κB, MAPK, and NLRP3 inflammasome pathways [11,12]. It has been demonstrated that the METTL3-dependent m6A regulation is responsible for the activation of the NLRP3 inflammasome pathway and pyroptosis resulting in inflammation and loss of alveolar bone in experimental periodontitis [13,14].

However, the biological role of m6A in periodontal tissues is not restricted to inflammatory destruction, as increasing evidence indicates that m6A regulation affects osteogenesis and periodontal regeneration [7,15,16]. The human periodontal ligament stem cells possess characteristics such as self-renewal and multilineage differentiation and are considered a major cell type for periodontal tissue regeneration, although their regenerative ability can be affected by inflammation in the periodontal environment [3,7]. M6A may influence the osteogenesis of periodontal ligament cells and stem cells via a range of downstream RNA targets and signaling pathways [7,15,16]. The m6A-mediated mechanism mediated by METTL3 affects the process of osteogenic differentiation via the stabilization or regulation of osteogenesis-associated RNAs, while the function of METTL14 is related to WNT3A-mediated osteogenesis signaling [7,16].

Current evidence indicates that the effects of m6A regulation in periodontitis are highly cell-, target-, and context-dependent, rather than being uniformly destructive or protective [7,17]. The identical regulator causes various biological effects based on the downstream RNA target and cell environment, where METTL3 increases osteogenic differentiation in some studies but is related to inflammation, pyroptosis, cellular senescence, and disturbance of bone remodeling in other research studies [12,14,16,17]. Such contradictory findings indicate the need to examine the interaction between individual m6A regulators and targets rather than considering m6A modification level globally as beneficial or detrimental [7,17].

Even though more evidence is rapidly accruing, there is still insufficient integration of the molecular mechanisms underlying the connection between m6A regulation and periodontal inflammation, destruction, and regeneration [18,19]. Therefore, this narrative review aims to synthesize current evidence summarizing existing evidence related to RNA m6A modification in relation to periodontitis, focusing on the role of m6A writers, erasers, and readers in inflammation, cell activity, osteogenesis, and bone remodeling. Particular attention is given to the context-dependent balance between m6A-mediated periodontal tissue destruction and regeneration, as well as to the potential therapeutic implications and directions for future research.

## 2. Materials and Methods

An electronic literature search was conducted on 19 August 2026 using MEDLINE (National Library of Medicine)-PubMed to identify studies investigating the role of N6-methyladenosine (m6A) RNA modification in periodontitis. The search strategy combined terms related to periodontitis with terms related to m6A modification and epitranscriptomic regulation. The following search query was used:

(“periodontitis”[Title/Abstract] OR “periodontal disease”[Title/Abstract] OR “periodontal inflammation”[Title/Abstract]) AND (“m6A”[Title/Abstract] OR “N6-methyladenosine”[Title/Abstract] OR “RNA methylation”[Title/Abstract] OR “epitranscriptomic*”[Title/Abstract])

The electronic search identified a total of 49 records. Titles and abstracts were initially screened to assess their relevance to the scope of the review. Potentially eligible studies were subsequently evaluated according to predefined inclusion and exclusion criteria. Following the screening and eligibility assessment, 34 original studies were included in the final review.

### Inclusion and Exclusion Criteria

The following inclusion criteria were applied:

1. Language: Articles published in English.
2. Topic: Studies investigating m6A RNA modification or m6A-related regulators in periodontitis, periodontal inflammation, or periodontal tissue and cellular homeostasis.
3. Type of study: Original research articles, including human, animal, in vitro, bioinformatic, transcriptomic, and epitranscriptomic studies.

The following exclusion criteria were applied:

1. Language: Articles published in languages other than English.
2. Topic: Studies not directly related to periodontitis or periodontal biology.
3. RNA modification: Studies focusing exclusively on RNA modifications other than m6A.
4. Type of publication: Review articles, systematic reviews, editorials, commentaries, and other non-original publications.
5. Insufficient relevance: Studies in which m6A modification was not sufficiently relevant to the main research question or periodontal outcomes.

**Figure 1:**
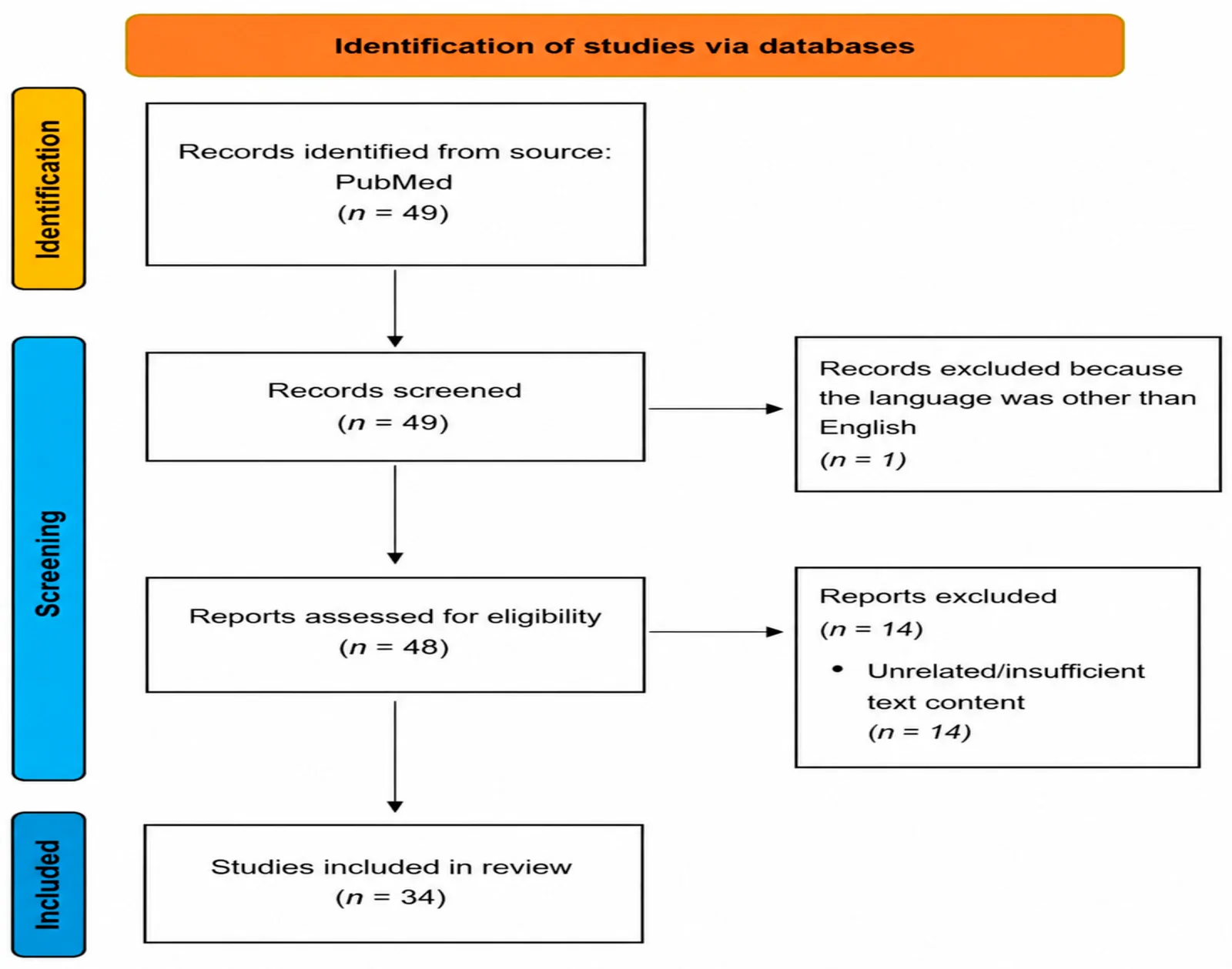
Display of the identification of studies via databases.

Figure 1 summarizes the process of identification of studies via databases.

## 3. Results

Table 1 summarizes the characteristics and key findings of the included studies. The main m6A-regulated molecular mechanisms identified across the included studies are summarized in Table 2. The available evidence indicates that m6A RNA modification is involved in various biological activities associated with periodontal disease, such as inflammatory signal transduction, inflammasome activation and pyroptosis, osteogenesis, osteoclastogenesis, cellular senescence, and osteoimmunology. These biological activities are regulated through m6A writers, erasers, and readers binding to particular RNA and signaling pathways. In addition, it should be noted that the biological effects of each m6A regulator depend on the context of the activity.

## 4. Discussion

The m6A modification of mRNA may regulate the cellular differentiation, development, and regulation of immune responses, through its effects on RNA splicing, transport, translation, and stability [39,40]. Dysregulation of m6A-mediated RNA regulation can impair normal cellular functions and contribute to the development of various human diseases, including cancer, metabolic disorders, and neurological diseases [39,40].

The available evidence indicates that m6A RNA modification contributes to molecular regulation, linking inflammatory signaling and immune responses with periodontal cell function and bone remodeling [8,9,18]. However, there is considerable variability in the strength of evidence in the available studies. Evidence in humans has been established only through transcriptomic and small observations [8,9,23], whereas molecular mechanism evidence has been established through in vitro studies and animals [11,13,14,16]. The results of transcriptomic and epitranscriptomic analysis of humans with periodontitis reveal changes in the expression of genes related to m6A and RNA modifications that correlate with immune cell infiltration and inflammation [8,9,21]. These findings provide evidence of disease-associated m6A alterations in human samples but do not by themselves establish causal molecular mechanisms. However, these observations should not be interpreted as evidence that an overall increase or decrease in m6A is inherently pathogenic, since mechanistic studies indicate that the biological outcome depends strongly on the specific regulator, RNA target, cell type, and inflammatory microenvironment [15,16,17,18]. Such dependence could be the explanation for the apparently opposing biological functions being assigned to the same m6A regulator under different experimental conditions [13,16,17].

A major point of convergence among recent experimental mechanistic studies is the involvement of m6A regulation in periodontal inflammatory signaling [11,13,14]. METTL3 and METTL14 can control SLC39A9 expression via m6A RNA methylation, which in turn affects intracellular Zn^2+^ levels and IL-6 secretion, and p38/JNK MAPK pathways mediate this inflammatory response [11]. Furthermore, METTL3-induced m6A RNA methylation increases the stability of CARD11 mRNA and induces NF-κB signaling [17]. Moreover, ALKBH5 can increase the stability of TLR9 mRNA via m6A methylation, which then activates MAPK pathways, promoting inflammatory signaling and suppressing osteogenic differentiation [33]. Additionally, the METTL3-regulated IRE1α pathway indicates the possibility of the impact of m6A on the interactions between the process of inflammation, osteogenic differentiation, and bone regeneration via another stress-related pathway [30]. Overall, these experimental findings suggest that modification by m6A does not work through one inflammatory pathway only, but rather acts as a higher-order regulator of multiple interconnected pathways in periodontal tissue damage [11,17,33].

This relationship is particularly evident in the regulation of the NLRP3 inflammasome and pyroptosis [13,14,36]. METTL3-mediated regulation of the TNFAIP3/NEK7/NLRP3 and NLRP3/IGF2BP3 pathways was related to inflammasome activation, the pyroptotic death of cells, inflammation, and the destruction of periodontal bone [13,14]. Moreover, the m6A reader protein ELAVL1 has been involved in the process of pyroptosis of periodontal ligament fibroblasts through the JAK2/STAT3/NLRP3 pathway [36]. The convergence of different m6A components on NLRP3-related mechanisms suggests that inflammasome regulation may link epitranscriptomic dysregulation with periodontal tissue damage [13,14,36]. Nevertheless, the relevance of these mechanisms across the different stages of periodontitis remains uncertain, since much of the mechanistic evidence is derived from stimulated cellular systems and experimental animal models [13,14].

The regulation of m6A is not only related to the promotion of periodontal tissue damage but also with osteogenesis and regeneration, which have been proven in several studies [15,16,25]. The activity of METTL3 was shown to promote osteogenic differentiation in PDLSCs by modulating the regulation of lncRNA4114, CUTALP/miR-30b-3p/Runx2, and miR-141-3p/ZEB1 pathways [3,22,25]. Similarly, METTL14-mediated WNT3A/β-catenin signaling and IGF2BP1-dependent regulation of BMP2 support the involvement of other components of the m6A machinery in periodontal osteogenesis and regeneration [7,37]. These findings are consistent with broader epitranscriptomic analyses showing substantial changes in the m6A landscape of periodontal ligament cells under inflammatory or mechanical conditions [9,10,15]. However, these regenerative mechanisms have been demonstrated predominantly in cellular and animal models and have not yet been established in clinical studies [3,7,16,25,37].

The apparently contradictory behavior of METTL3 is particularly informative and may represent one of the observations emerging from the current literature [13,16,17]. METTL3 can support osteogenic differentiation and osteoblast homeostasis through specific RNA targets, while under different inflammatory conditions it can promote NF-κB signaling, NLRP3-associated pyroptosis, and other processes favoring periodontal destruction [13,16,17,25]. In addition, METTL3/YTHDF2-dependent regulation of the FOXO3/SIRT1 pathway is responsible for development of oxidative stress and cellular senescence and impaired osteogenesis, while regulation of TP53BP1 by WTAP provides one more mechanism explaining how aberrant methylation impacts on the senescence of periodontal stem cells [34,35]. In contrast, METTL3-regulated LOR and DSC1 signaling has been associated with reduced inflammation, apoptosis and increased periodontal cell survival [31,32]. Collectively, these results indicate that the biological effects of individual m6A regulators are determined not by the regulator alone, but by several context-dependent factors, including the specific downstream RNA target, cell type, inflammatory environment, disease stage, and experimental model [16,17,31,34]. Such context-dependent regulation has important implications for the future development of therapies because the non-specific inhibition of METTL3 may not only inhibit unwanted signal transduction but also block any beneficial mechanisms [16,17,31].

Furthermore, the involvement of the m6A includes the regulation of the balance between bone formation, bone resorption, and immune modulation [18,24,26]. Experimental studies have implicated FTO in impaired BMSC osteogenesis through the SOST/Wnt pathway in diabetes-associated periodontitis and in osteoclastogenesis through FTO/YTHDF2-dependent regulation of Cyclin A2/CDK2 [24,26]. IGF2BP2 has also been linked to macrophage function and osteoclastogenesis through CD5L/CD36-related mechanisms, while WTAP-related changes in macrophage polarization provide additional evidence connecting m6A regulation with osteoimmune control of periodontal bone remodeling [18,28]. The function of IGF2BP2 is condition-specific, meaning that the results of regulation depend on the disease phase, and that time context should be considered [18]. Taken together, these observations indicate that m6A represents a molecular level at which inflammatory and skeletal processes interact in the case of periodontal disease [18,28].

The interaction between m6A and non-coding RNA networks further increases the complexity of this regulatory system [22,25,38]. In this regard, it is possible to refer to examples of regulation of the lncRNA4114 and CUTALP that provide m6A-dependent RNA stability, which, in turn, can impact downstream microRNAs and transcription networks regulating osteogenesis [22,25]. At the same time, METTL14/YTHDF2-dependent regulation of the NEAT1_2 has been associated with paraspeckles integrity and the YBX1-dependent transcription of IL-6, providing the connection between modification by m6A and nuclear structure and periodontitis-associated inflammatory signals [38]. Overall, based on the above examples, it can be stated that m6A must be considered an element of a regulatory network that includes both coding and noncoding RNAs [22,25,38].

From a translational perspective, m6A-related pathways may represent potential targets for future therapeutic development; however, the currently available evidence is predominantly preclinical [12,19]. According to experimental studies, modulation of some m6A-related pathways may stimulate osteogenic and periodontal regeneration processes, such as METTL3-dependent control of Per1 and m6A regulation of PDLSCs [12,19]. Nevertheless, the variable effects of certain m6A regulators based on the context demonstrate that possible therapeutic approaches should focus on certain regulator–RNA interactions rather than enhancement or inhibition of the overall m6A modification process [16,17,18]. Thus, more detailed research might be needed in order to determine the efficacy and safety of these approaches in humans.

m6A-related alterations may also have potential diagnostic relevance, although the available clinical evidence remains preliminary [8,9,23]. Transcriptomic studies have identified m6A-related signatures associated with periodontitis and different immune phenotypes, while human gingival and salivary analyses have identified candidate m6A-associated transcripts and modified nucleosides with potential discriminatory value [8,9,23]. However, these observations require validation in larger prospective cohorts, since several studies relied on relatively small clinical samples or secondary transcriptomic datasets [8,9,23]. Similarly, there is evidence linking the m6A-related genetic variant rs2723183 to IL-37 but the evidence in question was entirely computational without any direct verification in periodontal tissue [20]. Therefore, m6A-related molecules should be considered biomarker candidates rather than diagnostic indicators for periodontitis [8,9,20].

Conclusively, all available data confirms a scenario in which m6A modification acts as an interaction platform that connects periodontitis-related inflammation, cellular homeostasis and tissue regeneration, not being a one-way factor leading to diseases [8,15,18]. In certain conditions, m6A can participate in the processes such as inflammatory signaling, pyroptosis, senescence, osteoclast formation and bone resorption but in alternative conditions can ensure cellular survival, osteogenic differentiation, bone turnover and periodontal tissue regeneration [16,26,31,34]. Future studies should therefore move beyond measurements of global m6A abundance or isolated regulator expression and define specific regulator–RNA target–reader–cell type relationships using well-characterized human periodontal tissues and prospective clinical cohorts [8,9,17]. These studies will be needed to establish the translatability of the discovered experimental models into clinically valuable markers or therapies for periodontitis [9,18].

## 5. Limitations

This narrative review has several limitations. First, the literature search was limited to PubMed, and relevant studies indexed in other databases may therefore have been missed. In addition, no formal risk-of-bias or methodological quality assessment was performed. Although 34 original studies were included, considerable heterogeneity existed in study designs, experimental models, cell types, m6A regulators, and molecular targets. Furthermore, much of the available evidence remains preclinical or derives from bioinformatic and transcriptomic analyses, while direct evidence from human periodontal tissues and well-characterized clinical populations remains limited [8,9,18,20]. Therefore, further well-designed clinical and mechanistic studies are required before m6A-related biomarkers or therapeutic strategies can be translated into periodontal practice [8,9,19].

## 6. Conclusions

The m6A modification of mRNA is an epitranscriptomic mechanism that regulates periodontal inflammation, immune responses, cellular homeostasis, and bone remodeling [8,9,18]. Current evidence indicates that m6A regulators can contribute to both periodontal tissue destruction and regeneration, with their biological effects depending on the specific regulator, RNA target, cell type, and inflammatory microenvironment [13,16,17]. In particular, the involvement of m6A-related mechanisms in inflammatory signaling, pyroptosis, osteogenesis, osteoclastogenesis, and cellular senescence highlights their involvement in the pathogenesis and progression of periodontitis [14,26,34]. Although m6A-associated pathways may have potential therapeutic and diagnostic relevance, the current evidence remains predominantly preclinical, and further validation in human periodontal tissues and well-designed clinical studies is required before these findings can be translated into clinical practice [8,9,19].

## Figures and Tables

**Table 1:** Characteristics and key findings of the included studies (n = 34).

Study	Study Design	Sample/Model	m6A Component	Main Target/Focus	Key Findings
Lin et al., 2020 [20]	In silico genome-wide/GWAS analysis	Human periodontitis GWAS; 4910 European-American adults	m6A-associated SNPs	rs2723183–IL-37	1938 m6A-SNPs were identified, of which 104 were associated with periodontitis and 65 showed eQTL signals. rs2723183 emerged as a candidate variant potentially affecting m6A modification and expression of IL-37.
Zhang et al., 2021 [8]	Bioinformatic transcriptomic analysis	310 human periodontal samples; 241 periodontitis and 69 healthy	23 m6A regulators (writers, erasers, readers)	Immune microenvironment	Seventeen m6A regulators showed altered expression in periodontitis. Three distinct m6A modification patterns were identified and associated with differences in immune-cell infiltration, inflammatory responses, and HLA expression.
Ding et al., 2022 [21]	Bioinformatic transcriptomic analysis	Human gingival transcriptomic datasets; 427 periodontitis and 136 healthy samples	ALKBH5, FMR1, IGFBP3, RBM15B, YTHDF1, YTHDF2, ZC3H13	m6A regulator expression/periodontitis-associated signature	Seven m6A-related genes were identified. ALKBH5 was upregulated, whereas FMR1, IGFBP3, RBM15B, YTHDF1, YTHDF2, and ZC3H13 were downregulated in periodontitis.
Sun et al., 2022 [15]	Experimental epitranscriptomic study	Human periodontitis-derived and healthy PDLSCs exposed to static mechanical strain	METTL3; m6A-modified lncRNAs and mRNAs	Osteogenic differentiation/mechanical stress	143 lncRNAs and 739 mRNAs showed differential m6A methylation. METTL3 expression was reduced in periodontitis-derived PDLSCs, while METTL3 promoted their osteogenic differentiation.
Zhang et al., 2024 [22]	Experimental epitranscriptomic study	Human PDLSCs from 7 periodontitis patients and 10 healthy individuals	METTL3; m6A-modified lncRNAs and mRNAs	lncRNA4114/osteogenic differentiation	275 lncRNAs and 1292 mRNAs showed differential m6A modification, predominantly reduced in periodontitis-derived PDLSCs. METTL3 promoted osteogenic differentiation by increasing the stability of lncRNA4114.
Zou et al., 2023 [10]	Experimental epitranscriptomic study	Human periodontal ligament cells exposed to TNF-α and IL-1β	Transcriptome-wide m6A methylome	Inflammation/osteogenic differentiation	Inflammatory conditions markedly altered the hPDLC m6A landscape, with 5892 differentially methylated peaks (2636 hypermethylated; 3256 hypomethylated). Differential m6A modification was associated with histone-modifying genes, ECM organization, and impaired osteogenic differentiation.
Wang et al., 2023 [9]	Human epitranscriptomic study	Gingival tissues from 4 Stage III/IV periodontitis patients and 4 healthy controls	m6A mRNA epitranscriptomic microarray; MeRIP-qPCR	DNER, GNL2; PD-associated m6A landscape	458 differentially m6A-methylated transcripts were identified, predominantly hypomethylated (425 hypo vs. 33 hyper), together with 279 DMEGs. DNER and GNL2 showed reduced m6A methylation and expression in periodontitis and were validated as potential PD-related biomarkers.
Vignon et al., 2023 [23]	Human clinical epitranscriptomic biomarker study	Saliva from 16 periodontitis patients and 8 healthy controls	m6Am; m6A/m6Am–FTO axis	Salivary RNA modifications and free nucleosides	Multiplexed LC-MS/MS detected 16 RNA-derived and 27 free salivary nucleosides. Free m6Am was significantly decreased in Grade B periodontitis (*p* = 0.043), whereas inosine, cytidine and queuosine showed significant alterations. Significant nucleosides demonstrated AUC > 0.8, supporting their potential as salivary biomarkers.
Huang et al., 2024 [11]	Experimental mechanistic study	Human PDLCs; *P. gingivalis* LPS; murine ligature-induced periodontitis	METTL3, METTL14	SLC39A9–zinc–IL-6 axis	LPS increased global m6A and METTL3/METTL14 expression. METTL3/14 regulated SLC39A9 through m6A modification, influencing intracellular zinc and IL-6 production, with the involvement of p38/JNK MAPK signaling.
Zhou et al., 2023 [24]	Experimental mechanistic study	Murine diabetes-associated periodontitis; AGEs-treated BMSCs	FTO	SOST–Wnt signaling/osteogenesis	AGEs increased FTO and reduced m6A levels, impairing BMSC osteogenesis. FTO-mediated m6A demethylation stabilized SOST transcripts, whereas FTO knockdown or SOST silencing partially restored osteogenesis through Wnt signaling.
Ma et al., 2024 [18]	Experimental mechanistic study	Human periodontitis datasets/tissues; ligature-induced murine periodontitis; macrophages/osteoclasts	IGF2BP2	CD5L/CD36; inflammation and osteoclastogenesis	IGF2BP2 exhibited a stage-dependent dual role in periodontitis. Its depletion exacerbated inflammation and bone loss during early disease but reduced osteoclastogenesis and bone loss during advanced disease. IGF2BP2 directly interacted with Cd5l and Cd36 mRNAs, linking m6A reading to osteoclast differentiation.
Zhang et al., 2024 [16]	Experimental mechanistic study	LPS-stimulated osteoblasts; ligature-induced murine periodontitis	METTL3, YTHDF2	Dkk3/Sostdc1 regulation and osteoblast function	METTL3 promoted ribosome biogenesis, oxidative phosphorylation and osteoblast function through Wnt/β-catenin/c-Myc signaling. METTL3 regulated Dkk3 and Sostdc1 mRNA stability via YTHDF2, while METTL3 inhibition aggravated inflammation and alveolar bone loss in periodontitis mice.
Chen et al., 2024 [25]	Experimental mechanistic study	Human PDLSCs from healthy individuals and periodontitis patients	METTL3	lncRNA CUTALP and osteogenic differentiation	METTL3 was reduced in periodontitis-derived PDLSCs and promoted osteogenic differentiation. METTL3-mediated m6A modification enhanced lncRNA CUTALP stability and expression, which promoted osteogenesis through the miR-30b-3p/Runx2 axis.
He et al., 2024 [26]	Experimental mechanistic study (in vitro and in vivo)	RAW264.7 cells, mouse BMMs, and ligature-induced periodontitis mice	FTO; YTHDF2	Osteoclastogenesis and alveolar bone resorption	FTO enhanced the stability of Cyclin A2 and CDK2 mRNAs in a YTHDF2-dependent manner, promoting osteoclast precursor proliferation and inhibiting apoptosis. Pharmacological FTO inhibition with FB23-2 suppressed osteoclastogenesis and reduced alveolar bone loss in experimental periodontitis.
Zhou et al., 2024 [13]	Experimental mechanistic study (in vitro and in vivo)	Human gingival fibroblasts; ligature-induced periodontitis mice	METTL3	TNFAIP3/NEK7 and inflammasome activation	METTL3-mediated m6A promoted TNFAIP3 transcript degradation, reducing NEK7 ubiquitination and enhancing NLRP3 inflammasome activation and pyroptosis. Genetic or pharmacological METTL3 inhibition attenuated periodontal inflammation and alveolar bone loss.
Zhu et al., 2024 [12]	Experimental mechanistic study (in vitro and in vivo)	H_2_O_2_-induced oxidative stress in OMSCs; periodontal bone-defect model	METTL3 and m6A-related regulators	Per1 and periodontal bone regeneration	Quercetin altered the m6A landscape of OMSCs and increased METTL3 and m6A modification/expression of Per1, promoting osteogenic differentiation under oxidative stress. A quercetin-loaded bioglass injectable hydrogel enhanced periodontal bone repair through a Per1-dependent mechanism.
Deepika et al., 2024 [27]	Human case–control, in vitro and in silico study	Gingival tissues from 60 periodontitis patients and 60 healthy controls; Pg-LPS-stimulated HGFs	IGF2BP1	IL-17 signaling and periodontal inflammation	IGF2BP1 was significantly upregulated at the mRNA and protein levels in periodontitis tissues and Pg-LPS-stimulated HGFs. In silico analysis identified a putative m6A site in the IL-17 transcript, suggesting a potential m6A-dependent IGF2BP1/IL-17 regulatory axis in periodontal inflammation.
Li et al., 2024 [28]	Experimental mechanistic study (human scRNA-seq, in vitro and in vivo)	Human gingival scRNA-seq; Pg-LPS-stimulated BMDMs; BMSC co-culture; ligature-induced periodontitis mice	WTAP	Macrophage polarization and osteoimmune regulation	WTAP was upregulated in macrophages in periodontitis and associated with a pro-inflammatory M1 phenotype. WTAP knockdown reduced m6A levels, promoted M1-to-M2 polarization and enhanced BMSC osteogenic differentiation. Local AAV-mediated WTAP silencing promoted periodontal bone regeneration and reduced inflammatory cytokine expression in vivo.
Anand et al., 2025 [29]	Clinical case–control, in vitro and bioinformatic study	Gingival tissues from 35 periodontitis patients and 35 healthy controls; LPS-stimulated primary HGFs	METTL3	TREM-1-mediated periodontal inflammation	TREM-1 was upregulated in periodontitis gingival tissues. LPS stimulation increased global m6A levels, METTL3, TREM-1, IL-1β and IL-6 in HGFs. Predicted m6A sites within TREM-1 suggested a potential METTL3/m6A–TREM-1 inflammatory axis, although direct functional regulation was not experimentally validated.
Li et al. 2025 [30]	Experimental mechanistic study (in vitro and in vivo)	*P. gingivalis* LPS-induced periodontitis models	METTL3	IRE1α and periodontal inflammation	CUR-CD downregulated METTL3, reducing m6A modification and expression of IRE1α, while suppressing inflammation and promoting osteogenic differentiation and bone healing. IRE1α overexpression reversed these effects, supporting the involvement of the METTL3/m6A–IRE1α axis.
Li & Alimujiang, 2025 [3]	Experimental mechanistic study (in vitro)	LPS-treated human periodontal ligament stem cells (hPDLSCs)	METTL3	miR-141-3p/ZEB1 axis and osteogenic differentiation	LPS reduced METTL3 expression and impaired osteogenic differentiation. METTL3 overexpression increased m6A modification and DGCR8-mediated processing of pri-miR-141, thereby increasing miR-141-3p and suppressing ZEB1. Inhibition of miR-141-3p or ZEB1 overexpression partially reversed METTL3-mediated enhancement of osteogenesis.
Yang et al., 2025 [14]	Experimental mechanistic study (in vitro and in vivo)	STZ-induced diabetic mice with ligature-induced periodontitis; BMDMs and RAW264.7 macrophages exposed to *P. gingivalis* and high glucose; MC3T3-E1 osteoblasts	METTL3; IGF2BP3	NLRP3-mediated macrophage pyroptosis and alveolar bone loss	METTL3-mediated m6A modification enhanced NLRP3 mRNA stability through IGF2BP3, promoting macrophage pyroptosis, periodontal inflammation, and alveolar bone loss. METTL3 knockdown reduced pyroptosis and improved periodontal destruction and osteogenic activity in vivo.
Ma et al., 2025 [7]	Experimental mechanistic study	pPDLSCs from patients with periodontitis and hPDLSCs from healthy controls; GSE223924 human gingival dataset	METTL14 (writer)	WNT3A mRNA; osteogenic differentiation	METTL14 was downregulated in periodontitis-derived PDLSCs. METTL14 enhanced m6A modification and expression of WNT3A, promoting WNT3A/β-catenin signaling and osteogenic differentiation. WNT3A overexpression partially rescued the impaired osteogenesis induced by METTL14 knockdown.
Su & Chen, 2025 [31]	Clinical, bioinformatic and experimental mechanistic study (in vitro)	Gingival tissues from periodontitis patients and healthy controls; GSE10334 dataset; LPS-treated HPLFs	METTL3 (writer)	LOR-mediated inflammatory injury and fibroblast survival	METTL3 and LOR were downregulated in periodontitis. METTL3 enhanced m6A modification and stability of LOR mRNA, while LOR overexpression reduced inflammatory cytokine production and apoptosis and promoted HPLF proliferation and viability. METTL3 silencing partially reversed the protective effects of LOR overexpression.
Zhou & Wang, 2025 [17]	Clinical, bioinformatic, mechanistic in vitro and in vivo study	Periodontal ligament tissues from periodontitis patients (n = 31) and controls (n = 31); hPDLSCs; ligature-induced periodontitis rats	METTL3 (writer)	CARD11/NF-κB-mediated osteogenic differentiation and periodontal inflammation	METTL3 and CARD11 were upregulated in periodontitis. METTL3 promoted m6A modification and stability of CARD11 mRNA, activating NF-κB signaling and suppressing hPDLSC osteogenic differentiation. METTL3 knockdown enhanced osteogenesis and reduced inflammatory cytokines and periodontal bone loss in vivo.
Su & Chen, 2026 [32]	Clinical and experimental mechanistic study (in vitro)	PDL tissues from 16 periodontitis patients and 16 healthy controls; LPS-treated HPLFs	METTL3 (writer)	DSC1-mediated inflammation and cellular dysfunction	METTL3 and DSC1 were downregulated in periodontitis. METTL3 enhanced m6A modification and stability of DSC1 mRNA, while DSC1 overexpression reduced inflammatory cytokine production, NF-κB activation and apoptosis and improved HPLF survival. METTL3 knockdown partially reversed the protective effects of DSC1 overexpression.
Zhang et al., 2026 [33]	Clinical and experimental mechanistic study (in vitro)	Gingival tissues from 25 periodontitis patients and 10 healthy controls; LPS-treated hPDLSCs	ALKBH5 (eraser)	TLR9/MAPK-mediated inflammation and osteogenic differentiation	ALKBH5 and TLR9 were upregulated in periodontitis. ALKBH5 promoted TLR9 mRNA stability through m6A-dependent regulation, activating MAPK signaling and enhancing inflammation while suppressing osteogenic differentiation. ALKBH5 knockdown reduced inflammatory cytokines and restored osteogenic capacity, whereas TLR9 overexpression attenuated these effects.
Zhou et al., 2026 [34]	Experimental mechanistic study (in vitro and in vivo)	HFD/STZ + ligature-induced diabetic periodontitis mice; HG + *P. gingivalis* LPS-treated BMSCs	METTL3 (writer); YTHDF2 (reader)	FOXO3/SIRT1-mediated BMSC senescence and alveolar bone homeostasis	Hyperglycemic/inflammatory conditions increased METTL3 and m6A modification of FOXO3. METTL3-mediated m6A promoted YTHDF2-dependent FOXO3 mRNA decay, reducing FOXO3/SIRT1 signaling and promoting BMSC senescence, oxidative stress and impaired osteogenesis. METTL3 knockdown alleviated these effects, while SIRT1 activation rescued alveolar bone degeneration.
Xiong et al., 2026 [35]	Clinical, bioinformatic and experimental mechanistic study (in vitro)	Gingival tissues from 28 periodontitis patients and 20 healthy controls; periodontitis-derived PDLSCs	WTAP (writer-complex component)	TP53BP1-mediated PDLSC senescence and impaired osteogenic differentiation	WTAP was upregulated in periodontitis and promoted m6A-dependent stabilization and expression of TP53BP1 mRNA. WTAP knockdown reduced oxidative stress and senescence while enhancing osteogenic differentiation in P-PDLSCs. TP53BP1 silencing reversed WTAP-induced senescence, oxidative stress and osteogenic impairment.
Chen et al., 2026 [19]	Experimental in vitro and in vivo therapeutic study	PDLSCs; murine ligature-induced periodontitis model	METTL3/METTL14 (writers)	m6A-mediated osteogenic reprogramming of PDLSCs using a pH-responsive MXene nanoplatform	Delivery of METTL3/14 via MPDM enhanced PDLSC osteogenic differentiation and, together with ROS/cfDNA scavenging and antibacterial effects, reduced periodontal inflammation and alveolar bone loss in vivo.
Zhan et al., 2026 [36]	Experimental mechanistic study	*P. gingivalis* LPS-treated periodontal ligament fibroblasts; periodontitis model	ELAVL1 (reader)	JAK2/STAT3/NLRP3-mediated pyroptosis	ELAVL1 promoted periodontal ligament fibroblast pyroptosis and inflammatory responses through activation of the JAK2/STAT3/NLRP3 axis.
Wang et al., 2026 [37]	Experimental mechanistic study (in vitro and in vivo)	PDLSCs; LPS-*P. gingivalis*-induced periodontitis mice	IGF2BP1 (reader)	H3K18la/IGF2BP1/m6A/BMP2-mediated osteogenic differentiation and alveolar bone regeneration	IGF2BP1 was downregulated in periodontitis and positively regulated by H3K18 lactylation. IGF2BP1 recognized m6A-modified BMP2 and enhanced BMP2 mRNA stability, promoting PDLSC osteogenic differentiation. Increased lactylation and IGF2BP1 activity reduced periodontal inflammation and alveolar bone loss.
Du et al., 2026 [38]	Experimental mechanistic study (in vitro and in vivo)	Human PDLCs; periodontal ligament-targeted Mettl14-cKO mice with ligature-induced periodontitis	METTL14 (writer); YTHDF2 (reader)	NEAT1_2/YBX1-mediated periodontal inflammation	METTL14 was upregulated in periodontitis and promoted m6A modification and YTHDF2-dependent degradation of NEAT1_2. Loss of NEAT1_2 disrupted paraspeckles, releasing YBX1 to activate IL-6 transcription and exacerbate periodontal inflammation and alveolar bone loss. METTL14 deficiency was protective, whereas NEAT1_2 knockdown reversed this effect.

Note: “m6A component” refers to the m6A-related molecule or regulatory element investigated in each study, including m6A writers, erasers, readers, m6A-associated RNA modifications, and m6A-related genetic variants. “Main target/focus” indicates the principal molecular target, signaling pathway, or biological process investigated.

**Table 2:** Major m6A-regulated molecular mechanisms implicated in periodontitis.

Functional Axis	m6A Regulator(s)	Major Targets/Pathways	Biological Consequence	Representative Studies
Inflammatory signaling	METTL3/14	SLC39A9–Zn^2+^–IL-6; p38/JNK	Regulation of inflammatory cytokine production	[11]
Inflammatory signaling	METTL3; ALKBH5	IRE1α; TLR9/MAPK	Enhanced inflammatory signaling and impaired osteogenic responses	[30,33]
Inflammasome & pyroptosis	METTL3	TNFAIP3/NEK7/NLRP3; NLRP3/IGF2BP3	NLRP3 activation, pyroptosis and periodontal bone destruction	[13,14]
Pyroptosis	ELAVL1	JAK2/STAT3/NLRP3	Fibroblast pyroptosis and inflammatory response	[36]
Osteogenic differentiation	METTL3	lncRNA4114; CUTALP/miR-30b-3p/Runx2; miR-141-3p/ZEB1	Promotion of PDLSC osteogenic differentiation	[3,22,25]
Osteogenic differentiation	METTL14; IGF2BP1	WNT3A/β-catenin; BMP2	Enhanced osteogenesis and periodontal regeneration	[7,37]
Osteoblast function	METTL3/YTHDF2	Dkk3/Sostdc1–Wnt/β-catenin/c-Myc	Maintenance of osteoblast metabolism and bone homeostasis	[16]
Osteoclastogenesis	FTO/YTHDF2	Cyclin A2/CDK2	Osteoclast precursor proliferation and alveolar bone resorption	[26]
Diabetic periodontal bone loss	FTO	SOST/Wnt	Impaired BMSC osteogenesis under AGE exposure	[24]
Cellular senescence	METTL3/YTHDF2; WTAP	FOXO3/SIRT1; TP53BP1	Oxidative stress, cellular senescence and impaired osteogenesis	[34,35]
Fibroblast homeostasis	METTL3	LOR; DSC1	Reduced inflammation and apoptosis; enhanced periodontal cell survival	[31,32]
Osteoimmune regulation	WTAP; IGF2BP2	Macrophage polarization; CD5L/CD36	Regulation of macrophage phenotype, osteoclastogenesis and bone remodeling	[18,28]
lncRNA–paraspeckle signaling	METTL14/YTHDF2	NEAT1_2/YBX1/IL-6	Paraspeckle disruption, IL-6 transcription and periodontal inflammation	[38]
Regenerative/therapeutic regulation	METTL3; METTL3/14	Per1; m6A-mediated PDLSC reprogramming	Enhanced osteogenesis and periodontal bone regeneration	[12,19]

Note: “Functional axis” indicates the main biological process regulated by m6A-related mechanisms. “m6A regulator(s)” refers to the writer, eraser, or reader proteins involved in each mechanism. “Major targets/pathways” indicates the principal downstream RNA targets or signaling pathways, while “Biological consequence” summarizes the resulting cellular or periodontal effect. “Representative studies” lists the studies supporting each molecular mechanism.

## Data Availability

The original data presented in the study are included in the article; further inquiries can be directed to the corresponding author.
